# High-intensity focused ultrasound ablation enhancement in vivo via phase-shift nanodroplets compared to microbubbles

**DOI:** 10.1186/s40349-015-0029-4

**Published:** 2015-05-27

**Authors:** Linsey C. Moyer, Kelsie F. Timbie, Paul S. Sheeran, Richard J. Price, G. Wilson Miller, Paul A. Dayton

**Affiliations:** Joint Department of Biomedical Engineering, University of North Carolina-Chapel Hill and NC State University Campus, Box 7575, Chapel Hill, NC 27599 USA; Department of Biomedical Engineering, University of Virginia, Box 800759, Charlottesville, Virginia 22908 USA; Department of Radiology and Medical Imaging, University of Virginia, Box 801339, Charlottesville, Virginia 22908 USA

**Keywords:** Nanodroplet, Nanoemulsion, Phase-change contrast agent, Acoustic droplet vaporization, Focused ultrasound surgery, Ablation, Decafluorobutane, Dodecafluoropentane, Perfluorocarbon

## Abstract

**Background:**

During high-intensity focused ultrasound (HIFU) surgical procedures, there is a need to rapidly ablate pathological tissue while minimizing damage to healthy tissue. Current techniques are limited by relatively long procedure times and risks of off-target heating of healthy tissue. One possible solution is the use of microbubbles, which can improve the efficiency of thermal energy delivery during HIFU procedures. However, microbubbles also suffer from limitations such as low spatial selectivity and short circulation time in vivo. In this study, the use of a dual-perfluorocarbon nanodroplet that can enhance thermal ablation, yet retains high spatial selectivity and circulation half-life, was evaluated in vivo and compared to traditional microbubble agents during HIFU ablations of rat liver.

**Methods:**

High-intensity focused ultrasound (1.1 MHz, 4.1 MPa, 15-s continuous wave) was applied to rat liver in vivo, and heating was monitored during sonication by magnetic resonance thermometry. Thermometry data were analyzed to quantify temperature rise and ablated area, both at the target and prefocally, for HIFU applied 5, 15, or 95 min after intravenous injection of either nanodroplet or microbubble agents. Sham control experiments (no injected agents) were also performed.

**Results:**

At all three time points, nanodroplets significantly enhanced thermal delivery to the target, achieving temperatures 130 % higher and ablated areas 30 times larger than no-agent control sonications. Nanodroplets did not significantly enhance off-target surface heating. Microbubbles also resulted in significantly greater thermal delivery, but heating was concentrated at the proximal surface of the animal, causing skin burns. Furthermore, microbubbles resulted in lower thermal delivery to the desired target than even the control case, with the notable exception of the 95-min time point.

**Conclusions:**

Results indicate that the nanodroplet formulation studied here can substantially increase thermal delivery at the acoustic focus while avoiding prefocal heating. In contrast, microbubbles resulted in greater prefocal heating and less heating at the target. Furthermore, nanodroplets are sufficiently stable to enhance HIFU ablation in vivo for at least 1.5 h after injection. The use of a dual-perfluorocarbon nanodroplet formulation as described herein could substantially reduce HIFU procedure times without increasing the risk of skin burns.

## Background

High-intensity focused ultrasound (HIFU) can be used to non-invasively ablate tissue, both benign and malignant. HIFU is already FDA-approved for ablation of uterine fibroids, and pre-clinical evaluation of HIFU for the treatment of a variety of tumors, including breast, prostate, brain, pancreas, bone, and liver, is underway in the US, Europe, and Asia [1–4]. The clinical applications of HIFU are expanding, but safety is still a concern. Major obstacles associated with HIFU tumor ablation are superficial skin burns and long treatment times [5].

Microbubbles are known to increase the rate of HIFU ablation by reducing the acoustic energy required to cause heating and lesion formation [6]. However, microbubbles which are present outside the target region can lead to heating and/or ablation of healthy tissue outside the desired treatment area. Microbubbles also have a relatively short half-life in vivo [7], limiting the time over which they are effective during a HIFU surgical procedure. Because such procedures can last for several hours, an agent with a longer effective lifetime is highly desirable. Moreover, these micron-sized, lipid-shelled gas bubbles were originally designed as intravenously injectable ultrasound contrast agents, and as such, they remain confined to the vasculature due to their size. An ideal ablation-enhancing agent would be small enough to extravasate from blood vessels and accumulate in the target tissue. Nano-sized bubbles are particularly challenging to produce and exhibit resonance much higher than typical HIFU frequencies [8].

Due to the limitations of gas-filled microbubbles in HIFU applications, emulsions and droplets composed of various liquid perfluorocarbon agents have been proposed as alternative enhancers of HIFU ablation [9, 10]. Droplets composed of liquid perfluorocarbons can remain viable in circulation for substantially longer time periods than traditional gas-filled agents [11, 12]. Under sufficient negative pressure, droplets can be vaporized into microbubbles, a phenomenon often referred to as acoustic droplet vaporization [13]. The pressure required to convert liquid droplets into gaseous microbubbles depends on the size of the droplet and the type of perfluorocarbon utilized, as well as pressure and temperature in the medium [14–16]. Whether the droplets return to a liquid state or remain in the gaseous state also depends on similar parameters.

Much of the previous research on perfluorocarbon droplets and emulsions has centered on particles in the micron size range [13, 15, 17–19]; however, these particles are too large for extravasation. Nanoparticles less than about 200 nm are typically able to extravasate out of tumor vasculature due to the enhanced permeability and retention effect [20], giving them the potential to accumulate in tumor interstitium. Many of the liquid perfluorocarbon droplets described in previous studies have been composed of relatively high boiling-point perfluorocarbons in order to achieve stability [11, 15, 21, 22]. High boiling-point droplets typically require more acoustic energy to induce vaporization. The vaporization threshold of the droplets can be tailored by carefully selecting the ratio of perfluorocarbons [16, 23]. Previous studies in our lab have demonstrated that by combining two perfluorocarbons (decafluorobutane (C4F10) and dodecafluoropentane (C5F12)), the energy required to induce vaporization can be lowered while maintaining substantial stability (≥48 h) at body temperature [9, 24]. We have also demonstrated in vitro that these nanodroplets enhance HIFU ablation lesion formation at the target site whereas microbubbles lead to undesired surface lesion formation [9].

The goals of the study herein were to (1) evaluate the ability of our nanodroplet formulation to enhance HIFU ablation and (2) evaluate their effective lifetime in vivo. We hypothesized that our nanodroplet formulation would preferentially enhance HIFU ablation temperatures at the target location compared to microbubbles, while avoiding unwanted surface heating. Secondly, it was hypothesized that this enhanced thermal deposition could be induced over a longer time range following injection of nanodroplets compared to microbubbles. These hypotheses were tested in rat liver in vivo using magnetic resonance (MR) guidance and MR thermometry during HIFU ablation.

## Methods

### Microbubble and nanodroplet preparation

Microbubbles and nanodroplets were prepared in house. As previously described [24, 25], lipid-shelled microbubbles comprised of decafluorobutane cores were formed by mechanical agitation, resulting in 2.1 ± 0.5 μm diameter bubbles. Nanodroplet precursors were similarly formed by mechanical agitation but were comprised of a 1:1 ratio of decafluorobutane and dodecafluoropentane. These were then condensed under pressure and low temperature as previously described [24, 26] to result in 240 ± 65 nm diameter droplets. The concentration of each stock solution was approximately 1 × 10^10^ agents per milliliter. For the nanodroplet case, this estimate assumes direct conversion of the precursor microbubbles into droplets with 100 % efficiency. Both agents were diluted to 1/5 their original concentration in sterile saline prior to injection.

### MR-guided HIFU application in vivo

The effects of both perfluorocarbon microbubbles and nanodroplets during HIFU ablation were investigated in rat liver. The liver was selected because it is relatively homogeneous and sufficiently large to image with a clinical magnetic resonance imaging (MRI) scanner. All studies were approved by the University of Virginia’s Institutional Animal Care and Use Committee. Female Sprague-Dawley rats weighed between 165 and 210 g. The anterior side of the abdominal region was shaved and depilated on the day of the experiment. Rats were initially anesthetized through an intraperitoneal injection of Ketamine (40 mg/kg, Fort Dodge) and Dexdomitor (0.2 mg/kg, Pfizer) in sterilized water, and a catheter was placed in the tail vein. Rats were maintained in an anesthetized state using 12.5 mg/kg Nembutal administered through the tail vein catheter as needed. Microbubbles or nanodroplets were also administered through the tail vein catheter.

**Fig. 1:**
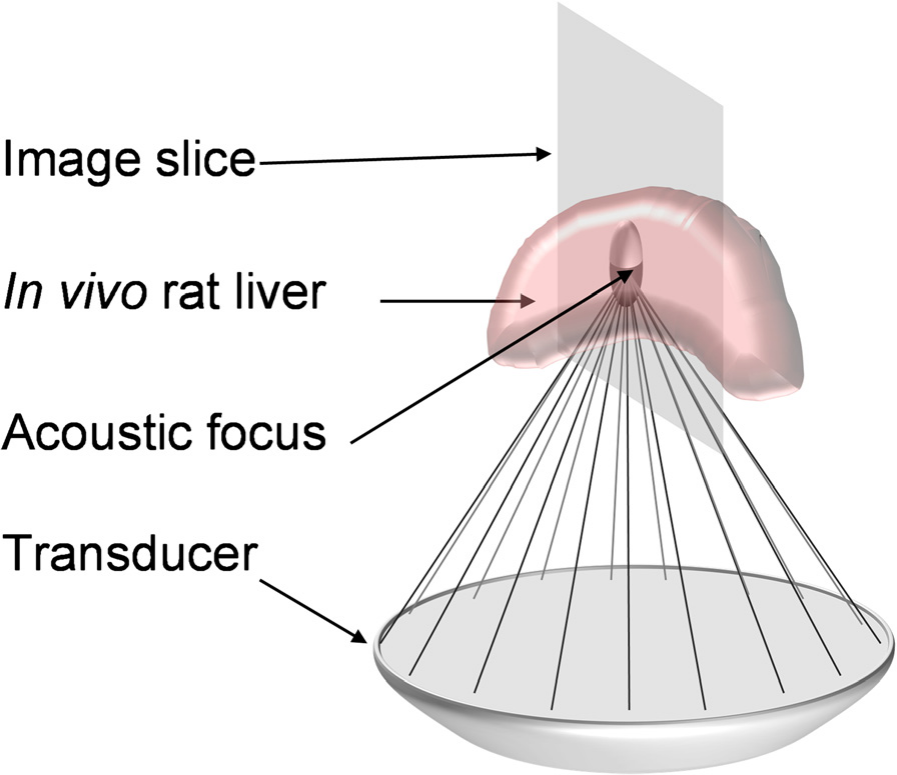
Schematic representation of the rat liver position with respect to the HIFU transducer. The acoustic focus was positioned ~7 mm inside the liver. Up to four separate HIFU sonications were performed in each liver. MR thermometry was performed in a vertical plane through the HIFU focus for each sonication

To investigate the location and degree of the thermal energy deposited by HIFU, sonication was performed using an MR-compatible HIFU system (RK-100, FUS Instruments Inc., Toronto, Ontario) while inside the bore of a clinical 3 T MRI scanner (Magnetom Trio, Siemens Healthcare, Malvern, PA). The spatial coordinates of the MRI and HIFU systems were synchronized at the beginning of each MR-guided HIFU session, by using an MR thermometry pulse sequence with high readout bandwidth to measure the location of a focal temperature rise induced in a hydrogel phantom. This measurement was performed at least twice, with the imaged slice oriented in vertical and horizontal planes, to locate the center of the focal spot along all three principal axes. Following this initial alignment procedure, the rat was positioned supine above the upward-facing HIFU transducer but inclined at a relatively steep angle with respect to horizontal to allow the vertical ultrasound beam to pass just below the ribs and into the liver (Fig. 1). A 2-in. square receive-only RF coil (FUS Instruments Inc.) was placed around the rat’s torso to obtain optimum MR signal from the region of interest. After positioning the animal, multi-plane MR images of the torso were acquired using a spoiled gradient-echo pulse sequence with high in-plane resolution (voxel size 0.33 × 0.33 × 2 mm^3^). These scout images were used to identify viable ablation sites with a clear acoustic path to the transducer unobstructed by ribs or bowel. Up to four target locations were chosen in each liver (depending on the available acoustic window), each centered at a depth of ~7 mm inside the proximal edge of the liver and all mutually separated by at least 5 mm.

HIFU was applied in conjunction with nanoparticles, microbubbles, or in the absence of either agent using a 1.14 MHz single-element transducer (75-mm diameter, *F*# = 0.8). For this transducer, the transverse and longitudinal dimensions of the acoustic pressure field are 1.5 and 7.6 mm (full-width at half maximum), respectively, at the ultrasound focus. Because the longitudinal half-width of the focal spot was nearly 4 mm, a target depth of 7 mm was chosen to ensure that the focal spot would be contained entirely within the liver. Each administration of nanodroplets or microbubbles consisted of a 0.l-ml dose of the diluted nanodroplet or microbubble solution described in the “Microbubble and nanodroplet preparation” section injected through the tail vein catheter, followed by a 0.3-ml heparinized saline flush. Each nanodroplet/microbubble HIFU application consisted of a 15-s continuous-wave sonication at 15 W of acoustic power (corresponding to 4.1 MPa peak negative pressure at the ultrasound focus). For comparison, sham control experiments were performed by applying 15-s sonications at 15 W in the absence of nanodroplets or microbubbles.

This acoustic power and duration were chosen based on a range of powers and durations that were explored previously in control animals and in tissue-mimicking polyacrylamide phantoms containing the same dual-perfluorocarbon nanodroplets used here [9]. In those phantom studies, it was found that peak negative pressures above 3 MPa were necessary to create ablation lesions. Moreover, ablation volume increased with both pulse length and pressure up to at least 20 s and 4 MPa, respectively. A 15-s HIFU exposure at 15 W (~4 MPa) was selected for the present in vivo study because this combination of duration and power was expected to be comfortably below the ablation threshold in control animals but comfortably above the ablation threshold in treated animals and thus represented a reasonable choice for demonstrating the potential advantages of such dual-perfluorocarbon agents.

MR thermometry was performed simultaneously with ultrasound application as described in the “MR thermometry and regional analysis” section below. To independently assess thermal enhancement provided by microbubbles and nanodroplets, only one HIFU application was performed at each target location (either before or after injection of one of these perfluorocarbon agents) and only one injection was performed in each rat. To assess the in vivo lifetime of these agents, HIFU was applied either 5, 15, or 95 min after a single injection. The total number of 15-s sonications performed across all rats in conjunction with each perfluorocarbon agent at each delay time is given in Table 1.

**Table 1:** Numbers of liver sonications performed for each type of agent at each time point

	Sham	Microbubbles			Nanodroplets		
Time after injection (min)	n/a	5	15	95	5	15	95
*N*	4	4	4	3	5	4	3

### MR thermometry and regional analysis

Dynamic MR thermometry was performed in conjunction with each sonication, by acquiring a time series of temperature-sensitive phase images of a single thin slice through the ultrasound focus using a spoiled gradient-echo pulse sequence. The measured phase-changes were then converted into temperature changes using the standard proton resonance frequency shift method [27, 28]. We were thereby able to monitor the temperature evolution at each pixel during HIFU application, as has been previously described in other thermometry studies [9]. Pulse sequence parameters for these thermometry scans included: echo time (TE) = 5.0 ms, repetition time (TR) = 26 ms, flip angle = 20°, readout bandwidth = 219 Hz/pixel, field of view = 72 × 96 mm^2^, matrix size = 96 × 128, slice thickness = 2 mm, in-plane resolution = 0.75 × 0.75 mm^2^, temporal resolution = 2.5 s per image. Images were interpolated by a factor of three in each dimension before temperature conversion, yielding 0.25 × 0.25 mm^2^ pixels in the temperature maps shown here.

In order to quantify the temperature rise at various penetration depths within the liver, the thermometry slice was oriented vertically, parallel to the direction of the HIFU beam (see Fig. 1). The horizontal position of this slice was centered on the transverse coordinates of the ultrasound focus. A high-resolution MR image of the same slice was also obtained and subsequently fused with each temperature map, to allow the anatomical location of focal heating to be more precisely determined. Pulse sequence parameters for these high-resolution scans included: TE = 3.1 ms, TR = 101 ms, flip angle = 50°, readout bandwidth = 322 Hz/pixel, field of view = 64 × 64 mm^2^, matrix size = 192 × 192, slice thickness = 2 mm, in-plane resolution = 0.33 × 0.33 mm^2^, 8 averages, total scan time = 2 min 35 s.

**Fig. 2:**
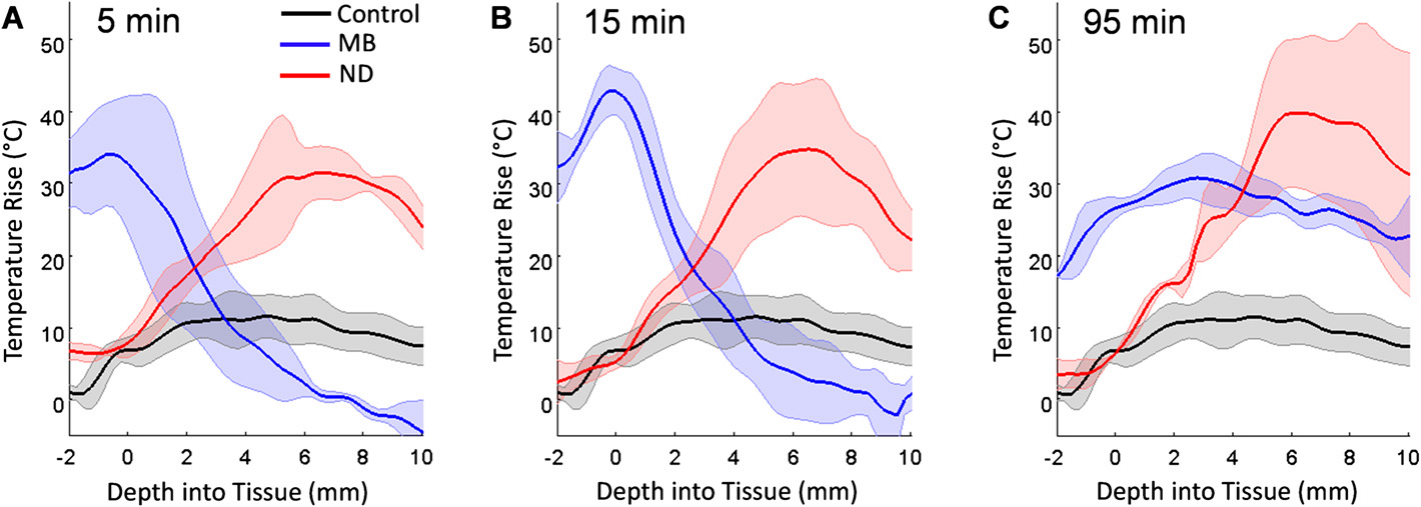
Temperature rise as a function of depth into the liver, calculated from the temperature maps. The heating profiles are plotted versus depth, with the *solid dark line* indicating the mean profile averaged over all sonications (*n* = 3, 4, or 5; see Table 1) performed at a given time point using a given agent and the *pale area* indicating a single standard deviation about the mean. Each plot shows the mean heating profile reached after 15 s of HIFU, applied **a** 5 min, **b** 15 min, or **c** 95 min following injection of microbubbles (*MB*), nanodroplets (*ND*), or the sham case (*Control*) where no agents were injected (this curve is the same in all three panels)

**Fig. 3:**
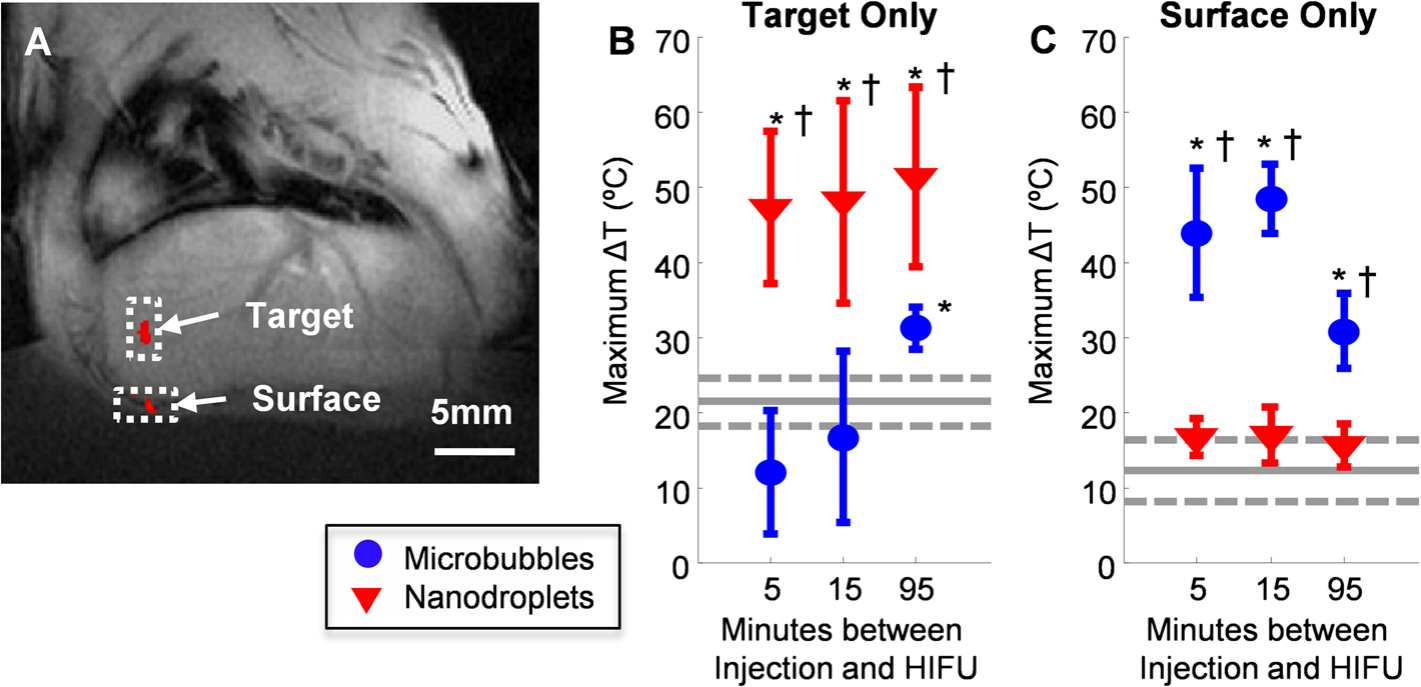
Maximum temperatures measured at the focal target and at the skin. **a** The temperature map during a control sonication was overlaid on the corresponding MR image of the rat including the liver. Two regions of interest were defined for quantitative regional analysis: **b** the target (focus of the HIFU beam), and **c** the surface of the animal. The *gray solid line* indicates the mean temperature change observed when HIFU was applied without an injection of perfluorocarbon agents (sham control experiments), and the *dotted lines* indicate one standard deviation. The *asterisk* indicates significance (*p* < 0.05) compared to the sham control results. The *dagger* indicates significance (*p* < 0.05) between microbubbles and nanodroplets. The data is displayed as mean ± S.D., where *n* = 3 to 5 (see Table 1)

Each temperature map was analyzed in three different ways in order to characterize and compare regional variations in focal heating for each sonication. In the first analysis, the temperature rise along the beam path was quantified as a function of depth into the liver (Fig. 2). This was accomplished by first locating both the horizontal position of the focused ultrasound target and the proximal margin of the liver by visual inspection of the high-resolution image. The average temperature rise was then computed over each 2.5-mm wide horizontal row of pixels above this margin, centered about the vertical line passing through the target location. In the second analysis, the degree of focal heating measured at the target depth was compared with that measured at the surface. This was accomplished by manually defining two 2.5 × 5.0 mm^2^ regions of interest, one centered at the known target location within the liver and the other centered on the interface between the skin and the proximal margin of the liver (see Fig. 3a). Thus, the second region of interest included pixels covering both the skin and liver near the surface of the animal. The average of the ten greatest pixel temperatures reached in each region of interest was calculated. In the third analysis, the ablated area at the target depth was compared with the ablated area at the surface of the liver. This was accomplished by counting the number of pixels within the same regions of interest described above, for which the measured temperature rise was at least 23 °C, and multiplying by the physical area covered by each pixel (0.0625 mm^2^). Reaching a tissue temperature of 60 °C, or approximately 23° above body temperature, is generally deemed sufficient to ensure ablation under the short sonication applied herein [4]. A two-tailed Student’s *t* test was applied to determine the statistical significance of measured differences between microbubbles, nanodroplets, and sham controls at each time point. A *p* value less than 0.05 was considered to be significant.

## Results

In the absence of nanodroplets or microbubbles, HIFU application for 15 s at 15 W was not sufficient to induce thermal ablation in the liver, whereas the same ultrasound parameters resulted in substantial tissue heating after intravenous administration of either nanodroplets or microbubbles. However, the spatial distributions of the thermal delivery were quite different in all three cases. The heating profile observed in the sham controls was relatively even over a range of tissue depths extending from about 1 to 10 mm, which is commensurate with the longitudinal extent of the ultrasound focus. In contrast, the heating profiles observed in the nanodroplet and microbubble cases revealed not only greater heating but more localized heating along the longitudinal beam axis. Nanodroplet-enhanced heating was concentrated over a range of tissue depths extending from about 4 to 10 mm (spanning the target depth of 7 mm), whereas microbubble-enhanced heating was concentrated over an even tighter range near the proximal edge of the liver. These data are summarized in Figs. 2 and 3.

The temperature rise measured as a function of depth into the liver, averaged over all same-agent HIFU sonications at a given time point, demonstrated that nanodroplets resulted in significantly more heating than the sham control at the target depth at all time points (5, 15, or 95 min after injection) (Figs. 2 and 3). The maximal temperature rise observed after nanodroplets had been injected was 51.5 ± 12.5 °C and occurred near the target [i.e., ~7 mm from the liver surface (see Fig. 2)]. This thermal enhancement was 2.3 times greater than the temperature rise reached in sham controls, and the corresponding ablated area was 31 times larger. The average maximum temperature rise reached in the sham control case was 22.4 ± 2.8 °C and occurred at the focal target region.

Surface heating was minimal in sham controls, only reaching 13.4 ± 3.6 °C. By comparison, microbubbles produced significant prefocal heating at the surface, which coincided with the presence of skin burns observed after the experiment (Figs. 2 and 3c). The temperature rise observed at the surface was up to 38° greater than that observed in the sham controls. In contrast, nanodroplets resulted in lower, albeit not statistically significant, heating at the surface compared to sham controls (Figs. 2 and 3c). At 5- and 15-min time points, microbubbles resulted in heating at the focus similar to sham controls. It was only at the 95-min time point that significant target heating was produced by HIFU in conjunction with microbubbles.

**Fig. 4:**
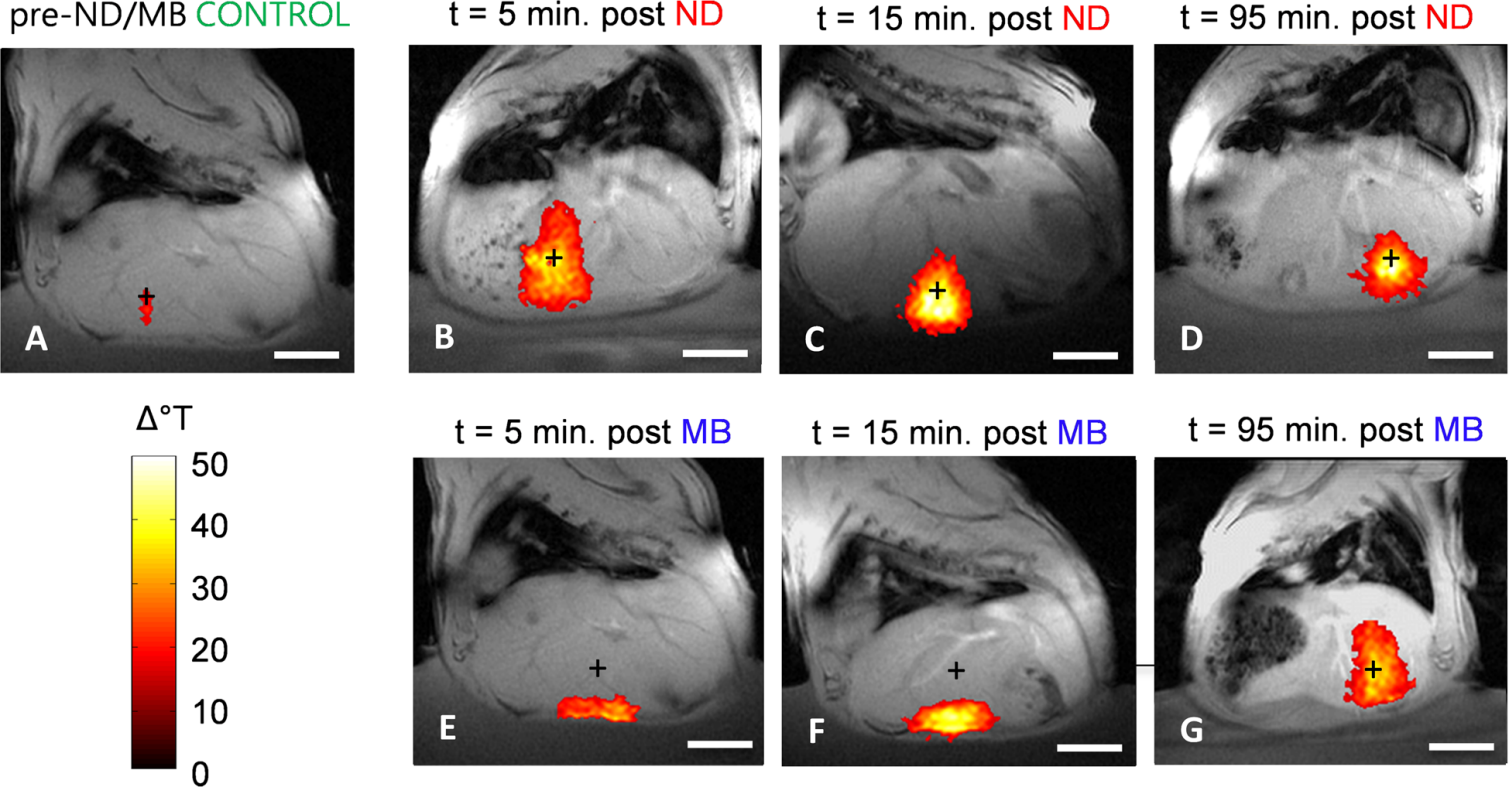
Example of MRI anatomical images with registered thermal maps. All temperature maps were thresholded at 15 °C before overlaying them onto the MR image. Images are representative MR images of rat livers acquired in a vertical plane passing through the ultrasound focus. **a** is a representative of the thermal profile observed before any agents were injected. **b**, **c**, and **d** are representatives of the thermal profiles 5, 15, and 95 min after the injection of nanodroplets (*ND*). **e**, **f**, and **g** are representatives of the thermal profiles 5, 15, and 95 min after the injection of microbubbles (*MB*). The *white scale bars* indicate 1 cm, and the *black crosshairs* indicate the intended target location

Representative thermal maps, each superimposed on its corresponding high-resolution anatomic image, are shown in Fig. 4. The color map threshold in these images only shows regions where the temperature rise reached at least 15 °C. These anatomically registered temperature maps clearly show the prefocal heating caused by the microbubbles at 5 and 15 min (Fig. 4e–f) compared to the focal heating produced by the nanodroplets (Fig. 4b–c).

**Fig. 5:**
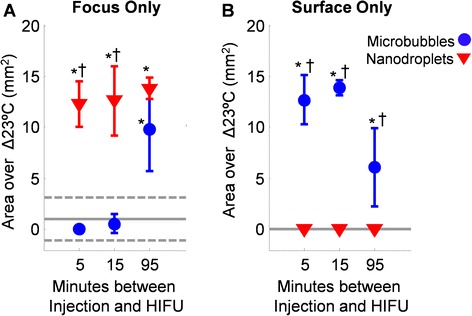
Ablation area measured at the focal target and at the skin. Ablation area was quantified by counting the number of image pixels wherein a temperature rise greater than or equal to 23 °C was measured by MR thermometry following 15 s of HIFU at 15 W. Ablation area was assessed **a** at the targeted region (acoustic focus) and **b** at the surface of the animal. The *gray solid line* indicates the mean ablation area when HIFU was applied without injection of any perfluorocarbon agents (sham control experiments), and the *dotted lines* indicate one standard deviation. The *asterisk* indicates significance (*p* < 0.05) compared to the sham control results. The *dagger* indicates significance (*p* < 0.05) between microbubbles and nanodroplets. The data is displayed as mean ± S.D., where *n* = 3 to 5 (see Table 1).

For our purposes here, we considered a temperature rise of 23 °C (corresponding to a nominal temperature of 60 °C in vivo) to be the threshold for thermal ablation. This criterion is in line with current clinical practice, in which the treatment goal is often to reach a temperature of at least 55–65 °C at the target, depending on the specific application [4, 29, 30]. According to this criterion, the nanodroplets resulted in significantly greater ablation areas (13.6 to 19.8 mm^2^ depending on the time point) at the focal target location compared to the sham control (1.0 ± 2.1 mm^2^, *p* < 0.01) at every time point investigated (Fig. 5). The ablation area at the surface was minimal (no pixels reached 23 °C or more) at each time point when either HIFU was applied alone (sham control case) or nanodroplets were present. In comparison, the microbubbles resulted in an ablation area at the target (0.0 to 0.6 mm^2^) that was not significantly different from the sham control at 5- or 15-min post injection. With microbubbles, a significant ablation area was achieved at the target only at the 95-min time point (see Fig. 5).

## Discussion

It is well known that microbubbles can enhance HIFU ablation lesion size and temperature rise; however, since microbubbles introduced intravascularly are present in most tissues not only at the acoustic focus but also in the near field, healthy tissues within the acoustic beam may also experience heating. Moreover, such prefocal energy deposition can prevent sufficient ultrasound energy from reaching the acoustic focus, effectively shielding the intended target from focal heating. Thus, although prefocal heating is an important safety consideration for all in vivo HIFU procedures [5], it is of special concern for microbubble-enhanced ablations. In this study, we demonstrated the advantage of a perfluorocarbon nanodroplet which remained acoustically inactive (did not enhance heating) unless exposed to sufficient acoustic pressure to phase-change into a gas. This allowed us to generate gas-filled bubbles only at the acoustic focus and thereby only achieve significant heating enhancement at the target.

Data illustrated that the use of microbubbles produced greater overall heating than sham controls, but that microbubble-enhanced heating at the HIFU parameters tested resulted in significant prefocal heating at both 5- and 15-min time points. In fact, each of the animals that underwent HIFU with microbubbles incurred skin burns. This was in stark contrast to the nanodroplet-enhanced HIFU case, where the nanodroplets produced minimal surface heating even though the focal target was only ~7 mm from the skin. Moreover, the use of microbubbles resulted in poor thermal delivery at the desired target, whereas nanodroplets resulted in significant thermal delivery enhancement at the acoustic focus. In preliminary no-agent, HIFU-only experiments, we found that substantially greater acoustic power (25 W) and twice as much time (30 s) was required to induce a thermal lesion in the liver. These results suggest that the nanodroplet formulation used here has the potential to expedite procedure times while avoiding surface heating associated with injected microbubbles.

The thermal profiles, maximum temperature rise, and ablated volumes all remained similar for each nanodroplet time point from 5- to 95-min post injection, indicating no loss in ablative properties during this time window. This result suggests that the nanodroplets remain acoustically active in vivo for at least 95 min in the liver. This longer timeframe would be conducive to enabling a single (or very limited number of) injection(s) for a full HIFU ablation surgery, which can take up to several hours to complete. However, it will be important for future studies to investigate the effective lifetime of these agents in other tissues including tumors.

One observation worth noting is that although prior studies indicated microbubble circulation half-lives in rats is on the order of minutes [7], our studies demonstrated that microbubbles contributed to enhanced HIFU ablation in the liver 95 min after injection. Although lesion size and temperature rises were generally smaller than those produced by the nanodroplets at this time point, this result was still surprising as it was not anticipated that microbubbles would still be acoustically active after such a long time period. This data suggests that the lipid encapsulated microbubbles were somehow preserved in the liver, possibly either by Kupffer cell phagocytosis [31] or due to mechanical capture within liver sinusoids [32]. The observation that the depth of microbubble-induced temperature rise shifted away from the surface and toward the focal target at later time points further suggests that prefocal shielding effects become greatly reduced as microbubbles are cleared from circulation.

By extension, this observation also suggests that prefocal shielding might be reduced at earlier time points by decreasing the number of microbubbles administered in the initial injection. However, previous in vitro studies by our group indicated that significant prefocal heating could be avoided only by using very low microbubble concentrations, for which there was also relatively little thermal enhancement at the target [9]. Hence, whereas the performance of microbubbles in vivo might be improved somewhat by carefully optimizing the injected concentration and sonication parameters, we suspect that one would still not be able to achieve the level of robustness and control possible with a phase-change contrast agent. Such agents are designed to activate only above a certain pressure threshold, which makes it relatively easy to design a robust sonication protocol that produces enhanced heating at the ultrasound focus but avoids unwanted heating elsewhere. Furthermore, because the activation threshold of the nanodroplets is theoretically independent of the agent concentration, their overall performance should be relatively insensitive to both the initially injected concentration and its variation over time.

A limitation of the present study is that our in vivo rat liver model did not permit sonication at appreciable tissue depths, due to both the small size of the animal and the limited sonication window accessible between the between ribs and bowel. Thus, we were unable to explore larger target depths more in line with eventual clinical applications. Nonetheless, there is good reason to expect that nanodroplet-enhanced sonications would perform as well or better at deeper target locations. Although greater ultrasound power would certainly be necessary at greater target depths, in order to overcome the additional beam attenuation and keep the focal pressure above the activation threshold, the potential for prefocal activation at the skin should actually be smaller, since the ultrasound pressure field is much smaller further from the focus.

Finally, it is also worth noting that phase-change nanodroplets can be formulated with identical excipients to FDA-approved microbubbles, which might be advantageous to the eventual clinical translation of such nanodroplet technology.

## Conclusion

We have shown that HIFU-mediated liver ablation can be significantly enhanced with mixed perfluorocarbon nanodroplets compared to HIFU alone. In addition, data suggested that although microbubbles could also enhance HIFU ablation, they resulted in unintended prefocal thermal delivery and skin burns. These experiments highlight the benefit of an agent to enhance HIFU delivery that converts to a microbubble only at the acoustic focus, such as a phase-change agent. Furthermore, data supports the argument that an agent with an activation threshold tuned to match the delivered focal acoustic pressure can provide a mechanism for keeping thermal delivery enhancement to a spatially selected region of interest, as determined by the pressure field and vaporization threshold of the agent. From these data, we conclude that phase-change nanodroplets may potentially make MR-guided focused ultrasound surgery safer and shorten procedure times by enhancing ablation speed and volume. Future studies will investigate the ability of these nanodroplets to enhance HIFU ablation in vivo in a tumor model.

## Abbreviations

HIFU: high-intensity focused ultrasound
MR: magnetic resonance
MRI: magnetic resonance imaging
